# Phytosterols protect against diet-induced hypertriglyceridemia in Syrian golden hamsters

**DOI:** 10.1186/1476-511X-13-5

**Published:** 2014-01-06

**Authors:** Todd C Rideout, Vanu Ramprasath, John D Griffin, Richard W Browne, Scott V Harding, Peter JH Jones

**Affiliations:** 1 Department of Exercise and Nutrition Sciences, University at Buffalo, Buffalo, NY 14214, USA; 2 Richardson Centre for Functional Foods and Nutraceuticals, University of Manitoba, Winnipeg, Manitoba R3T 2 N2, Canada; 3 Biotechnical and Clinical Laboratory Sciences, University at Buffalo, Buffalo, NY 14214, USA; 4 Diabetes and Nutritional Sciences Division, School of Medicine, King’s College London, London SE1 9NH, UK

**Keywords:** Phytosterols, Ezetimibe, Triglycerides, Lipogenesis

## Abstract

**Background:**

In addition to lowering LDL-C, emerging data suggests that phytosterols (PS) may reduce blood triglycerides (TG), however, the underlying mechanisms are not known.

**Methods:**

We examined the TG-lowering mechanisms of dietary PS in Syrian golden hamsters randomly assigned to a high fat (HF) diet or the HF diet supplemented with PS (2%) for 6 weeks (n = 12/group). An additional subset of animals (n = 12) was provided the HF diet supplemented with ezetimibe (EZ, 0.002%) as a positive control as it is a cholesterol-lowering agent with known TG-lowering properties.

**Results:**

In confirmation of diet formulation and compound delivery, both the PS and EZ treatments lowered (p < 0.05) intestinal cholesterol absorption (24 and 31%, respectively), blood non-HDL cholesterol (61 and 66%, respectively), and hepatic cholesterol (45 and 55%, respectively) compared with the HF-fed animals. Blood TG concentrations were lower (p < 0.05) in the PS (49%) and EZ (68%)-treated animals compared with the HF group. The TG-lowering response in the PS-supplemented group was associated with reduced (*p* < 0.05) intestinal SREBP1c mRNA (0.45 fold of HF), hepatic PPARα mRNA (0.73 fold of HF), hepatic FAS protein abundance (0.68 fold of HD), and *de novo* lipogenesis (44%) compared with the HF group. Similarly, lipogenesis was lower in the EZ-treated animals, albeit through a reduction in the hepatic protein abundance of ACC (0.47 fold of HF).

**Conclusions:**

Study results suggest that dietary PS are protective against diet-induced hypertriglyceridemia, likely through multiple mechanisms that involve modulation of intestinal fatty acid metabolism and a reduction in hepatic lipogenesis.

## Background

Although LDL-C levels have decreased among US adults in recent years largely due to the widespread and effective use of lipid lowering medication, hypertriglyceridemia is increasingly prevalent with 33.1% of Americans having borderline high triglyceride (TG) levels [1]. This mean increase in TG concentrations amongst men and women over the past 20 years has coincided with the alarming obesity trend and is further associated with increased risk of acute pancreatitis and cardiovascular disease (CVD) [2]. Data from the National Health and Nutrition Examination Survey (NHANES) suggests that > 80% of overweight (BMI 25–30 kg/m^2^) and obese (BMI ≥30 kg/m^2^) subjects have TG concentrations ≥1.69 mmol/L [3].

Moderate hypertriglyceridemia (1.69-2.24 mmol/L) is treated with body weight reduction through lifestyle modifications of diet and physical activity while severe cases (>5.6 mmol/L) require first-line drug therapy with a range of pharmaceuticals including statins, fibrates, niacin, and prescription n-3 fatty acids [4]. Marine-derived n-3 fatty acids are considered the most effective nutraceutical TG-lowering option (~30%), however, phytosterols (PS) have recently been explored for their potential TG-lowering effects beyond their established LDL-C lowering efficacy. Although the majority of clinical PS intervention studies have failed to observe significant TG lowering responses, two studies specifically designed to examine the TG-lowering potential of PS reported reductions in the range of 11-27%, depending on baseline TG concentration [5,6]. The precise mechanism (s) responsible for the TG-lowering effects of PS are not known, however, we recently observed an increase in fecal saturated fatty acid excretion in C57Bl6 mice fed a PS-enriched diet, suggesting a possible interference with intestinal fat absorption [7]. Furthermore, although previous work suggests that PS may be effective in reducing TG in subjects with established, overt hypertriglyceridemia, it is unknown if PS provides protection against diet-induced increases in TG following high fat feeding. Considering the effectiveness of PS in reducing LDL-C by interfering with intestinal cholesterol absorption, knowledge of the TG-lowering potential of PS and the underlying mechanisms involved will be critical in ascertaining the utility of PS as a potential therapy against mixed dyslipidemia. Therefore, the objective of this study was to examine the effectiveness of PS in protecting against diet-induced hypertriglyceridemia in Syrian golden hamsters fed a high fat TG-raising diet. We utilized EZ as a comparative control as it is another well-characterized cholesterol-absorptive inhibitor that is recognized to reduce circulating TG concentrations in humans and a previous study using the Syrian golden hamster [8].

## Results

### Establishment of diet-induced dyslipidemic model

Table 1 demonstrates whole-body growth and blood lipid endpoints in response to the consumption of the HF versus the LF diet. HF feeding for six weeks increased (p < 0.05) final body weight and blood lipid concentrations including total-C, HDL-C, non-HDL-C, and TG. Although plasma aspartate transaminase (AST) did not differ (p > 0.05) between the two groups, plasma alanine aminotransferase (ALT) was higher (p < 0.05) in the HF versus the LF group, suggesting a potential hepatic inflammatory response to HF feeding.

**Table 1:** **Establishment of high fat feeding model on body weight and blood lipid parameters in Syrian golden hamsters**^**2**^

	**Diets**	
**Item**	**LF**^**1**^	**HF**^**1**^
*Whole body parameters*		
Initial body weight (g)	124.13 ± 1.81	126.21 ± 1.81
Final body weight (g)	139.58 ± 3.27	151.18 ± 3.42*
Body weight change (%)	15.18 ± 2.64	25.75 ± 2.75*
*Blood biochemistry*		
Total cholesterol (mmol/L)	4.65 ± 0.31	6.45 ± 0.31*
Non-HDL cholesterol (mmol/L)	2.19 ± 0.21	2.87 ± 0.21*
HDL-cholesterol	2.45 ± 0.21	3.57 ± 0.21*
Triglycerides (mmol/L)	2.48 ± 0.46	5.26 ± 0.46*
Glucose (mmol/L)	10.76 ± 0.66	12.16 ± 0.87*
*AST (U/L)*	90.18 ± 11.92	86.20 ± 8.90
*ALT (U/L)*	62.0 ± 8.24	83.60 ± 8.32*

^1^LF, low fat control diet; HF, High fat diet.

^2^mean ± SEM, n = 12.

*Significantly different from LF (*p* < 0.05).

### Blood lipid and sterols

**Figure 1:**
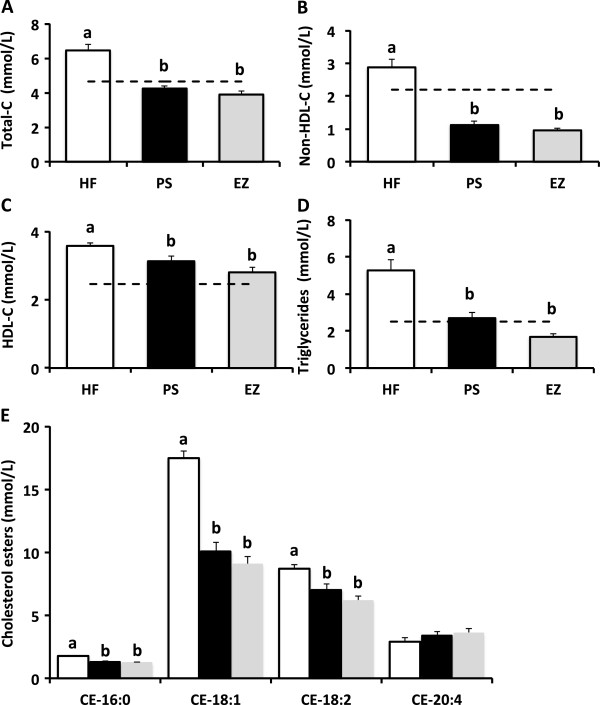
**Blood lipid response to phytosterol and ezetimibe therapy in Syrian golden hamsters. (A)** total cholesterol (mmol/L); **(B)** non-HDL cholesterol (mmol/L); **(C)** HDL-cholesterol (mmol/L); **(D)** triglyceride (mmol/L); **(E)** Plasma cholesterol esters (nmol/L) including cholesteryl palmitate (CE-16:0), cholesteryl oleate (CE-18:1), cholesteryl-linoleate (CE-18:2) and cholesteryl arachidonate (CE 20:4). ^ab^Groups not sharing a superscript are significantly different (*p* < 0.05). The dashed line in A, B, C and D represent the mean lipid response following consumption of the LF diet.

**Figure 2:**
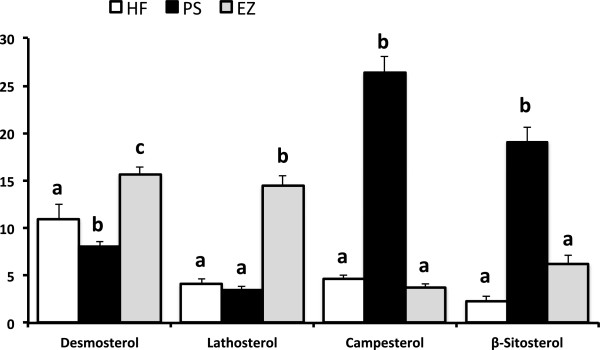
**Red blood cell phytosterols (nmol/g) in response to phytosterol and ezetimibe therapy in Syrian golden hamsters. **^ab^Groups not sharing a superscript are significantly different (*p* < 0.05).

As expected, total, non-HDL-C, and HDL-C were lower in the PS (34, 61, and 12.5%) and EZ (39, 66, and 21.6%) groups versus the HF-fed animals with no difference between the PS and EZ groups (Figure 1A, B, C). Furthermore, blood TG concentrations were lowered (*p* < 0.05) to a similar extent with PS (49%) and EZ (68%) therapy compared with the HF group (Figure 1D). Dietary PS and EZ supplementation resulted in similar reductions (*p* < 0.05) in plasma concentrations of CE-16:0, CE-18:1, and CE-18:2 versus the HF group (Figure 1E). PS feeding led to an expected increase (*p* < 0.05) in RBC campesterol and sitosterol concentrations compared with the HF and EZ groups (Figure 2) but also reduced (*p* < 0.05) RBC desmosterol concentrations compared with the other groups. EZ supplementation increased (*p* < 0.05) RBC desmosterol and lathosterol concentrations compared with the HF and PS groups (Figure 2).

### Hepatic cholesterol and fatty acids

**Figure 3:**
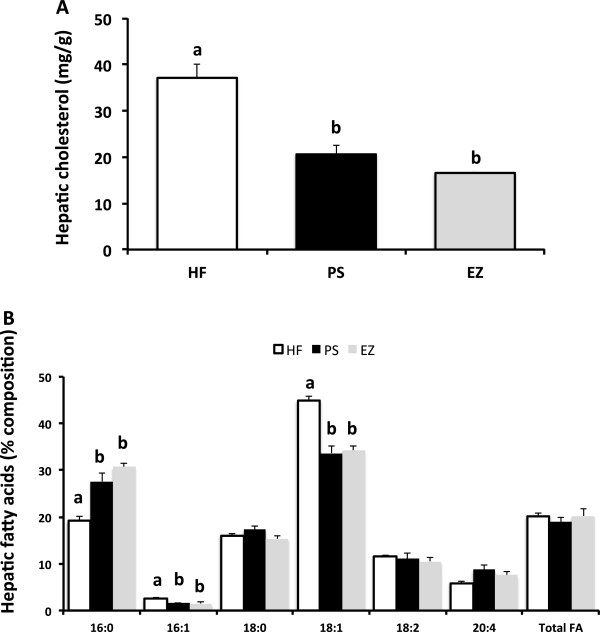
**Hepatic cholesterol (A, mg/g) and fatty acid (B, % composition) in response to phytosterol and ezetimibe therapy in Syrian golden hamsters. **^ab^Groups not sharing a superscript are significantly different (*p* < 0.05).

Hepatic cholesterol was lowered similarly in the PS (45%) and the EZ (55%) groups compared with the HF-fed animals (Figure 3). No difference in total hepatic fatty acids was observed, however, PS and EZ treatment produced in a similar shift in hepatic fatty acid composition by increasing 16:0 (~50%) and reducing 16:1 (~40%) and 18:1 (~24%) compared with the HF-fed animals (Figure 3).

### Stable isotope analyses

**Figure 4:**
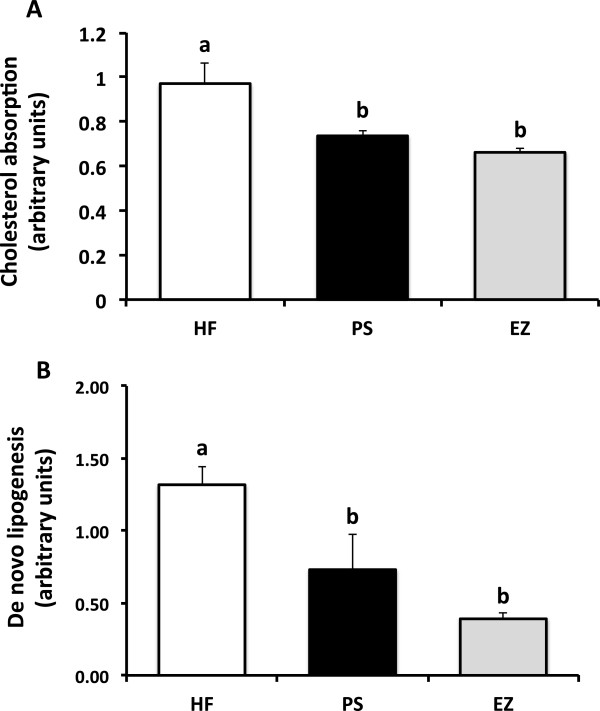
**Cholesterol absorption (A) and *de novo *lipogenesis (B) in response to phytosterol and ezetimibe therapy in Syrian golden hamsters. **^ab^Groups not sharing a superscript are significantly different (*p* < 0.05).

Cholesterol absorption was lowered (p < 0.05) to a similar extent in the PS (27%) and EZ (37%) groups, compared with the HF group (Figure 4). Compared with the HF group, hepatic *de novo* lipogenesis was lowered (*p* < 0.05) by feeding PS (44%) and EZ (70%) with no difference between the PS and EZ groups.

### mRNA and protein expression

**Figure 5:**
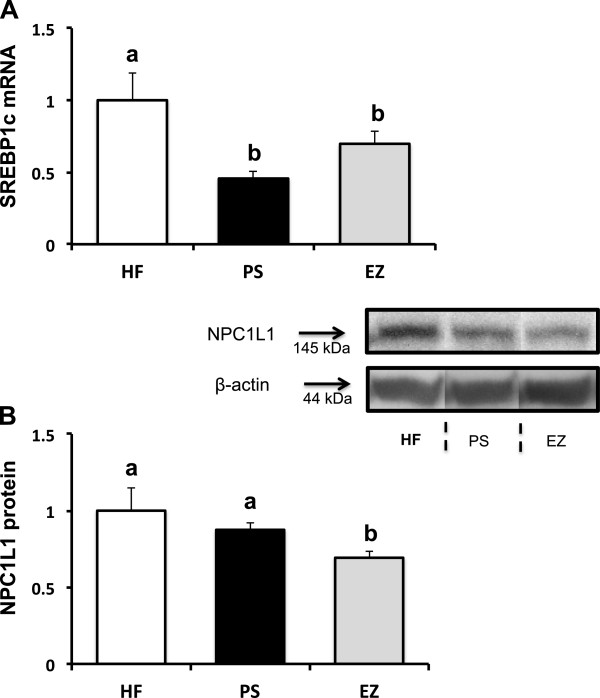
**Intestinal mRNA and protein expression following phytosterol and ezetimibe therapy in Syrian golden hamsters. (A)** SREBP1c mRNA and **(B)** NPC1L1 protein abundance. All genes normalized to the HF group and expressed relative to β-actin. ^ab^Groups not sharing a superscript are significantly different (*p* < 0.05).

No changes in intestinal NPC1L1, FABP1, or CD36 were observed in response to PS or EZ versus the HF group (data not shown). Compared with the HF group, SREBP1c mRNA expression was lowered (*p* < 0.05) in response to PS (0.45 fold of HF) and EZ supplementation (0.69 fold of HF, Figure 5A). However, EZ treatment lowered NPC1L1 protein abundance (0.69 fold of HF) compared with the HF and PS groups (Figure 5B).

**Figure 6:**
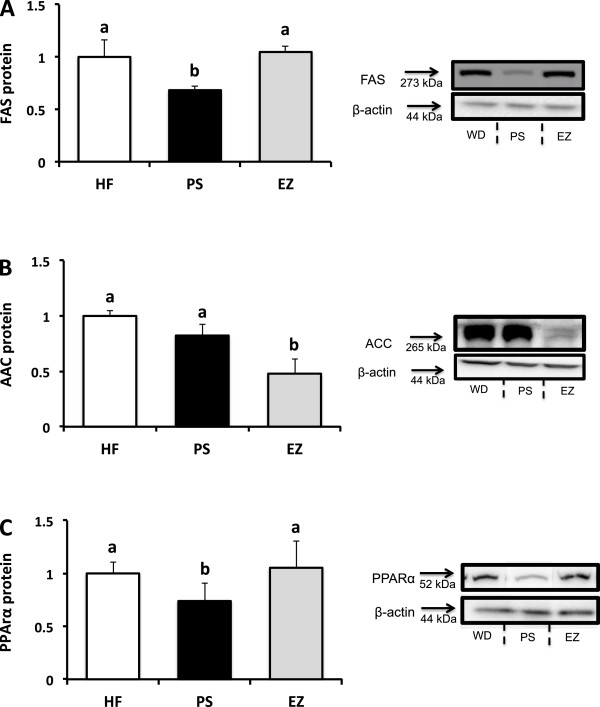
**Hepatic protein abundance following phytosterol and ezetimibe therapy in Syrian golden hamsters. (A)** FAS protein abundance; **(B)** ACC protein abundance; **(C)** PPARα protein abundance. All proteins normalized to the HF group and expressed relative to β-actin. ^ab^Groups not sharing a superscript are significantly different (*p* < 0.05).

Compared with the HF group, hepatic FAS protein abundance was lowered (0.68 fold of HF, *p* < 0.05) with PS consumption but was not affected by EZ therapy (Figure 6A). EZ lowered hepatic ACC protein abundance (0.47 fold of HF, *p* < 0.05) compared with the HF group, but was not affected by PS supplementation (Figure 6B). Compared with the HF group, PPARα protein abundance was lowered (0.73 fold of HF, p < 0.05) in the PS group but not affected by EZ supplementation (Figure 6C).

## Discussion

Findings from this study suggest that dietary PS, in addition to their established cholesterol-lowering properties, may also protect against diet-induced hypertriglyceridemia. This TG-lowering response appears to be due to modulation of fat metabolism in the intestine and the liver as evidenced by a reduction in intestinal SREBP1c and PPARα mRNA expression and a decrease in hepatic FAS protein abundance and *de novo* lipogenesis. These results would seem to confirm the limited animal [9,10] and human data [5,11] that suggests a TG-lowering response to PS supplementation and suggest that PS may be effective in protecting against diet-induced mixed dyslipidemia.

In this diet-induced hypertriglyceridemic hamster model, blood and tissue lipid responses to PS supplementation were comparable to those observed with EZ, a suitable positive control as it has a similar cholesterol-lowering effect to PS but also possesses a more well-characterized effect on TG metabolism including reductions in blood TG [12-15] and amelioration of hepatic TG accumulation [16] and steatosis [17,18]. As a confirmation of both the diet composition and experimental protocol, both PS and EZ-treated animals demonstrated an expected inhibition of intestinal cholesterol absorption and a reduction in blood total and non-HDL-C cholesterol below that of the LF-fed animals. Although it is not completely understood how a cholesterol-absorptive inhibitor could modulate TG metabolism, a few recent reports have examined the issue specifically with EZ.

Naples et al. (2012) suggested that EZ may lower postprandial TG by modulating intestinal fatty acid absorption and chylomicron assembly by reducing apoB48 production and modulating the expression of intestinal lipid synthesis and transport genes in fructose and fat-fed hamsters [19]. Similarly, we observed a reduction in intestinal SREBP1c mRNA in both the EZ and PS groups, thus confirming the previous report by Naples et al. (2012) and further suggesting that PS may reduce blood TG through a similar intestinal SREBP1c-related mechanism. It is not known why we did not observe a corresponding reduction in the cytoplasmic or nuclear protein abundance of SREBP1c in the PS and EZ groups. We previously reported a reduction in intestinal PPARα mRNA expression and increased fecal FA loss in C57BL/6 J mice fed a PS supplemented diet [7]. Taken together, it appears that at least part of the TG-lowering effects of PS is associated with the modulation of multiple intestinal FA regulatory targets.

With the knowledge that excessive hepatic cholesterol is lipogenic through the activation of the liver X-receptor (LXR) [20,21], a more direct link between the cholesterol-lowering properties of EZ and TG-reductions has been proposed. In a HF-fed hamster model, Ushio et al. (2013) reported that in response to a limitation in intestinal cholesterol absorption, EZ reduced hepatic oxysterol LXRα ligands, thus inhibiting LXRα-induced transcriptional stimulation of SREBP-1c [17]. However, after failing to observe a reduction in SREBP1c lipogenic targets in the EZ-treated animals, the authors concluded that EZ did not likely reduce hepatic lipogenesis through this pathway. Contrary to this conclusion, our results suggest that EZ reduces ACC protein abundance and inhibits hepatic *de novo* lipogenesis. Our results further suggest that PS supplementation may modulate hepatic fat metabolism through a similar reduction in hepatic lipogenesis, albeit through a reduction in the protein abundance of FAS. Further work is required to determine if this reduction in hepatic lipogenesis in PS-supplemented animals is directly related to the observed reduction in hepatic cholesterol concentration and an associated inhibitory effect on the LXRα-SREBP1c lipogenic stimulus pathway. The observed reduction in hepatic lipogenesis in PS-supplemented hamsters is in direct contrast to our previous report of increased lipogenesis in C57BL/6 J PS-supplemented mice [7]. Given the similarity in the design factors between the two studies, including diet, feeding protocol and stable isotope analysis, this discrepancy highlights the underlying differences in lipid metabolism amongst rodent species and cautions against premature translation of biological efficacy and mechanistic data regarding potential disease treatment strategies from animal models [22].

Although hepatic fatty acid profile is a major determinant of *de novo* lipogenesis, this does not appear to be a contributing factor to explain the reduced fatty acid synthesis observed in the PS and EZ treated animals. Both fatty acid chain length and degree of unsaturation affect lipogenic gene expression, with monounsaturated fatty acids (MUFA) and longer-chain polyunsaturated fatty acids (PUFA) having a general inhibition and saturated fatty acids (SFA) having a stimulatory effect in parallel with fatty acid elongation pathways [23]. As both PS and EZ both shifted the hepatic FA profile towards an increase in palmitate (~50%) and a reduction in oleic acid (~24%), it appears that modulation of hepatic fatty acid distribution was not a determinant in the observed reduction in lipogenesis observed in these groups. Although little work has examined hepatic FA concentration in response to PS, our results are supported by a previous report by Brufau et al. (2006) suggesting an increase in the hepatic SFA fraction in guinea pigs supplemented with PS [24].

Intracellular hepatic fatty acids also serve as lipid signaling molecules by inducing PPARα activation. In this way, PPARα is thought to protect against fat accumulation by enhancing expression of rate-limiting enzymes in the β-oxidation pathway. Previous work suggests that a wide spectrum of fatty acids are capable of transactivating PPARα and inducing DNA binding activity, particular long chain PUFA [25]. However, as we observed no change in hepatic total fatty acids and a reduction in 18:2 in the PS-supplemented group, the underlying mechanisms and physiological implication of reduced PPARα abundance is unclear.

Although inclusion of EZ as a comparative control to examine the TG-lowering mechanisms of PS may be considered a study strength, direct comparison of the lipid-lowering magnitude between the PS and EZ groups may not be appropriate given that the dietary inclusion levels, although similar to those typically used in previous animal studies, exceeds normal PS intakes (2 g/d) and EZ prescribed doses (~10 mg/d) in humans. Although the cholesterol-lowering response to PS (~10%) and EZ (~20%) are fairly consistent, the magnitude of TG lowering is considerably more variable for both PS (6-15%) and EZ (10-20%) and likely dependent on baseline TG concentrations [11,26-28].

## Conclusions

Here, we demonstrate that PS supplementation offers protection against diet-induced hypertriglyceridemia in Syrian golden hamsters fed a high fat diet. Our data suggest that several mechanisms, namely reductions in dietary fat absorption, intestinal SREBP1c mRNA expression, hepatic PPARα abundance, and de novo lipogenesis may be contributing to the reduction in circulating TG. Clarification of the mechanisms underlying these TG reductions may broaden the clinical utility of PS supplementation beyond LDL-C lowering to a potential therapy for mixed dyslipidemias.

## Methods

### Animals and diet

The animals used in this experiment were cared for in accordance with the guidelines established by the Canadian Council of Animal Care [29]. All procedures were reviewed and approved by the Animal Care Committee at the University of Manitoba (protocol number F06-013/1/2). Forty-eight male Syrian golden hamsters were acquired from Charles Rivers and brought to the Animal Model Research Facility at the Richardson Centre for Functional Foods and Nutraceuticals at the University of Manitoba. Hamsters were housed individually in cages with shavings in a temperature-controlled room (20°C) with a 12 h light/dark cycle and had free access to water. At the initiation of the experiment, hamsters were randomly assigned to 1 of 3 treatment diets (n = 12) for 6 weeks according to Table 2: (1) High fat diet (HF, AIN 76A Western Diet); (2) HF diet supplemented with 2% PS (Reducol, Forbes Meditech); (3) HF diet supplemented with 0.002% EZ [8] (Merck/Schering-Plough Pharmaceuticals). An additional group of animals was maintained on a low fat diet (LF, AIN76) to establish the effects of the HF feeding on obesity, and hepatic and plasma lipid response.

**Table 2:** Formulation of experimental diets (%) for Syrian golden hamsters

	**Diets**^**1**^			
**Ingredient**	**LF**	**HF**	**PS**	**EZ**
Sucrose	12	34.1	34.1	34.1
Milk fat	3.7	19.96	19.96	19.96
Casein	19.5	19.47	19.47	19.47
Maltodextrin	10	9.99	9.99	9.99
Corn starch	43.2	4.99	4.99	4.99
Phytosterol mix^2^	-	-	2	-
Ezetimibe^3^	-	-	-	0.002
Powdered cellulose	5	4.99	2.99	4.99
AIN76-A mineral mix	3.5	3.49	3.49	3.49
AIN76-A vitamin mix	1	0.99	0.99	0.99
Corn oil	1.2	0.99	0.99	0.99
Calcium carbonate	0.4	0.39	0.39	0.39
DL-methionine	0.3	0.29	0.29	0.29
Choline bitartrate	0.2	0.19	0.19	0.19
Cholesterol	-	0.15	0.15	0.15
Ethoxyquin	0.001	0.005	0.005	0.005
Total	**100**	**100**	**100**	**100**
Energy (%)				
Fat	11.8	40.0	40.0	40.0
Carbohydrate	69.1	44.2	44.2	44.2
Protein	19	15.8	15.8	15.8

^1^LFD, low fat/low cholesterol control; HF, high fat diet; PS, plant sterol; EZ, ezetimibe.

^2^Reducol, Forbes Meditech (Campestanol 7% w/w; Stigmasterol <1% w/w; sitosterol 71% w/w; sitostanol 15% w/w).

^3^Merck/Schering-Plough Pharmaceuticals.

### Sample collection and processing

Following the 6-week feeding period, hamsters were anesthetized with isoflurane for blood and tissue collection then killed by exsanguination while in the surgical plane of anesthesia. Fasting (8 hrs) blood (serum and EDTA plasma) was collected by cardiac puncture and processed as previously described [7]. The liver and proximal small intestine were quickly excised, rinsed/flushed in chilled saline (pH 7.4, 154 mM containing 0.1 mM phenylmethylsulfonyl fluoride) and flash frozen in liquid nitrogen. All tissues were stored at −80°C until further processing and analyses.

### Blood lipid and sterol analyses

Plasma total cholesterol (TC), high-density lipoprotein cholesterol (HDL-C), non-HDL cholesterol (non-HDL-C), and TG were determined by automated enzymatic methods on a Vitros 350 chemistry analyzer (Ortho-Clinical Diagnostics, Markham, Ontario, Canada). Non-HDL-C was calculated by difference.

Serum free cholesterol and cholesteryl esters were measured by high performance liquid chromatography (HPLC) according to our modification of Vercaemst et al. [30]. Briefly, one volume of serum was diluted with five volumes of isopropanol containing cholesteryl heptadecanoate as the internal standard. Samples were vortexed for 30 minutes, centrifuged, and injected (50 uL) onto a 25 cm × 4.6 mm reverse phase column using an isocratic mobile phase of acetonitrile/isopropanol (60:40 v/v) at a flow rate of 2.0 mL/min and 45°C. Unesterified cholesterol and cholesteryl esters [palmitate (CE-16:0), oleate (CE-18:1), linoleate (CE-18:2), and arachidonate (CE-20:4)] were detected by their UV absorbance at 208 nm and quantified in comparison to pure standards after correcting for internal standard recovery.

Red blood cell PS concentrations were determined by gas–liquid chromatography according to previously established procedures [31] using a gas chromatography (6890 GC, Agilent Technologies, Palo Alto, California) equipped with flame ionization detector and auto-injector system. A 30-m SAC-5 column (Sigma-Aldrich Canada Ltd., Oakville, Ont.) was used. Briefly, 5-α cholestane as an internal standard was added to each of the samples followed by addition of methanolic potassium hydroxide and saponification. Sterols were extracted from the mixture with petroleum ether. Extracted samples were derivatized with TMS reagent (pyridine-hexamethyldisilazan-trimethylchlorosilane (9:3:1, v/v)) and samples were injected into the GC. The injector and detector were set at 300 and 310 degrees C, respectively. The flow rate of the carrier gas, helium was 1.2 ml/min with the inlet splitter set at 100:1. Individual PS were identified using authentic standards (Sigma-Aldrich Canada Ltd., Oakville, Ont). Internal standards were used to calculate detector response factors. Campesterol and β-sitosterol concentrations were determined by identifying the peak sizes and expressing them relative to 5-α cholestane internal standard.

### Hepatic sterol and fatty acid analyses

Hepatic cholesterol was extracted and analyzed according to our previously published procedures [7,32]. Approximately 500 mg of pulverized liver was spiked with α-cholestane as internal standard and saponified in freshly prepared KOH–methanol at 100°C for 1 h. The non-saponifiable sterol fraction was extracted with petroleum diethyl ether and dried under N_2_ gas. For analysis of hepatic fatty acids, approximately 0 · 5 g of pulverized liver was spiked with heptadecanoic acid (C17:0) as internal standard. Total lipids were isolated from liver tissue with a modified Dole mixture (3 hepatane:12 propanol:3 DDH_2_O, vol:vol) followed by extraction with heptane: DDH_2_O (3:1 vol:vol) [33]. Fatty acid extracts were methylated with methanolic boron trifluoride (Sigma Aldrich, St. Louis, MO).

Sterol and fatty acid fractions were analyzed using a Shimadzu GC-17A gas chromatograph fitted with a flame ionization detector. A SAC-5 capillary column (30 m × 0 · 25 mm × 0 · 25 mm, Supelco, Bellefonte, CA, USA) was used for cholesterol analyses. Fatty acid methyl esters were separated using a Supelcowax 10 column (30 m × 0 · 25 mm with 0 · 25 m film thickness; Supelco, Bellefonte, PA, USA). Relative hepatic fatty acid content was calculated by using individual FA peak area relative to the total area and expressed as the percentage of total fatty acids.

### Intestinal RNA preparation and real-time RT-PCR

Total RNA was isolated from whole intestinal tissue using TRIzol reagent (Invitrogen Canada Inc., Burlington, ON). RNA concentration and integrity was determined with spectrophotometry (260 nm) and agarose gel electrophoresis, respectively. RNA preparation and real-time RT-PCR was conducted using a one-step QuantiTect SYBR Green RT-PCR kit (Qiagen Inc., Mississauga, ON, Canada) on a Biorad MyiQ real time PCR system according to previously established protocols [34]. Sequences of sense and antisense primers for target and housekeeping genes were based on previously published reports for NPC1L1 [35], CD36, FABP2 [36], SREBP1c, [37], and β-actin [38].

### Immunoblot analysis of intestinal and hepatic regulatory proteins

Immunoblots were prepared as previously described [34]. Nuclear and cytoplasmic extracts for immunoblot analyses of peroxisome proliferator-activated receptor alpha (PPARα, SC-9000, Santa Cruz Biotechnology), SREBP1c (Novus Biologicals, NB600-582), fatty acid synthase (FAS, C2OG5, Cell Signaling), and acetyl-CoA carboxylase (ACC, C83B10, Cell Signaling), were separated using the CelLytic™ NuCLEAR™ extraction kit (Sigma, Saint Louis, Missouri, USA). Intestinal apical membrane extracts were extracted according to a previously established protocol probed for NPC1L1 (Santa Cruz, sc-67237) [39]. Target proteins were normalized to β-actin and quantified using Image J (National Institutes of Health, Bethesda, Maryland).

### Stable isotope analyses

Hamsters were given an intraperitoneal injection of deuterium (100 μl) 2-hours prior to euthanization. Lipogenesis rates (%/day) were quantified using the uptake rate of deuterium from body water into newly synthesized hepatic-palmitate extracts over 2 h at the end of the feeding experiment [40,41]. Deuterium enrichment of hepatic-palmitate was quantified using an on-line gas chromatography/combustion/isotope ratio mass spectrometry approach (Agilent 6890 N chromatograph interfaced with a Thermo Delta V Plus isotope ratio mass spectrometer (Bremen, Germany). Isotope abundance, expressed in delta (δ) per mil (‰), was calculated in hepatic-palmitate and plasma water (precursor pool) using H_2_ as a reference gas and further corrected against the international reference, Standard Mean Ocean Water (SMOW). *De novo* lipogenesis rates were calculated with the following equation:

$$\begin{array}{l} \mathrm{De}\;\mathrm{novo}\;\mathrm{lipogenesis}\;\left(\%/\mathrm{day}\right)\\ \;=\Delta \mathrm{TGFA}\;(‰)/\Delta \mathrm{plasma}\;(‰)\times 0.477*100 \end{array}$$

Where ΔTGFA is the change in deuterium enrichment in hepatic-palmitate; Δplasma is the change in the deuterium enrichment of the precursor plasma water; and 0.477 is derived from 0.87 g-atom ^3^H per g-atom carbon incorporated into adipose tissue fatty acids and a correction factor to account for the glycerol moiety as previously described [42]. Lipogenesis rates are expressed relative to the HF group.

Forty-eight hours prior to euthanization, hamsters were given an oral gavage of safflower oil containing 5 mg of [3,4]-^13^C cholesterol (99% enriched; CDN Isotopes). As an indicator of cholesterol absorption, GC-combustion-isotope ratio MS (Delta V Plus, Thermo Scientific) was used to determine the ^13^C enrichment (^13^C/^12^C ratio) of free cholesterol in RBC compared with the non-enriched hamster RBC ^13^C-cholesterol enrichment over 48 h [43].

### Statistical analyses

Data were analyzed with a general linear model ANOVA using experimental block as a fixed factor [44]. To establish the effect of HF-feeding, responses between the LF versus the HF groups were compared using a paired t-test. Multiple comparisons between treatment groups were analyzed with Tukey’s post-hoc test. Data were analyzed with SPSS 16 for Mac (SPSS Inc, Chicago IL). Data are presented as mean ± SEM. All results are the means from 12 animals unless otherwise stated. Differences were considered significant at p ≤ 0.05.

## Competing interests

The authors declare that they have no competing interests.

## Authors’ contributions

TCR designed and conducted the research, analyzed the data, and wrote the initial draft manuscript; VR conducted the research; JG conducted the immunoblot and RNA analyses; RWB conducted the lipid analyses; SVH conducted the stable isotope analysis; PJ designed the research. All authors read and approved the final manuscript.
